# Involvement of P2X_7_ Receptor in Proliferation and Migration of Human Glioma Cells

**DOI:** 10.1155/2018/8591397

**Published:** 2018-01-09

**Authors:** Zhenhua Ji, Yuting Xie, Yu Guan, Yujian Zhang, Kin-Sang Cho, Min Ji, Yongping You

**Affiliations:** ^1^Department of Neurosurgery, The First Affiliated Hospital with Nanjing Medical University, Nanjing 210029, China; ^2^Department of Head and Neck Surgery, Affiliated Tumor Hospital of Nantong University, Nantong 226001, China; ^3^Department of Ophthalmology, Affiliated Hospital of Nantong University, Nantong 226001, China; ^4^Schepens Eye Research Institute, Massachusetts Eye and Ear, Department of Ophthalmology, Harvard Medical School, Boston, MA 02114, USA

## Abstract

Previous studies have demonstrated that activation of P2X_7_ receptors (P2X_7_R) results in the proliferation and migration of some types of tumor. Here, we asked whether and how the activated P2X_7_R contribute to proliferation and migration of human glioma cells. Results showed that the number of P2X_7_R positive cells was increasing with grade of tumor. In U87 and U251 human glioma cell lines, both expressed P2X_7_R and the expression was enhanced by 3′-O-(4-benzoylbenzoyl) ATP (BzATP), the agonist of P2X_7_R, and siRNA. Our results also showed that 10 *μ*M BzATP was sufficient to induce the proliferation of glioma cell significantly, while the cell proliferation reached the peak with 100 *μ*M BzATP. Also, the migration of U87 and U251 cells was significantly increased upon BzATP treatment. However, the number of apoptotic cells of U87 and U251 was not significantly changed by BzATP. In addition, the expression of ERK, p-ERK, and proliferating cell nuclear antigen (PCNA) protein was increased in BzATP-treated U87 and U251 glioma cells. PD98059, an inhibitor of the MEK/ERK pathway, blocked the increased proliferation and migration of glioma cells activated by BzATP. These results suggest that ERK pathway is involved in the proliferation and migration of glioma cells induced by P2X_7_R activation.

## 1. Introduction

Malignant glioma is the most common malignant tumor of the central nervous system [1]. The invasive performance and easy transformation from lower grades to higher grades were the main causes of the poor prognosis of glioma [2, 3]. Low apoptosis and aggressive cell proliferation, invasion, and angiogenesis of glioma make it very challenging to be treated [4, 5]. Therefore, investigating the underlying mechanisms of glioma malignant proliferation and invasive growth was essential to glioma treatment.

Microenvironment of solid tumors is characterized by a strikingly features of high concentration of adenosine and adenosine triphosphate (ATP) [6]. Activated purine receptor has been shown in many kinds of tumors [7]. However, its role in tumorigenesis is not fully elucidated. Purinergic receptors have been studied extensively in inflammation and degeneration of the central nervous system in recent years [8–10]. P2X_7_R is a nonselective cation channel receptor [11]. Brief exposure to P2X_7_R agonist such as ATP or 3′-O-(4-benzoylbenzoyl) ATP (BzATP) leads to the opening of cation channel allowing K^+^ efflux and Ca^2+^ and Na^+^ influx into the cells [12]. However, prolonged activation of P2X_7_R will be resulted in formation of large aqueous pores and finally leads to cell death [13]. Compared to all other members of P2X family and other ligand-gated ionotropic receptors, the most striking feature of P2X_7_R mediated currents is the absence of current desensitization with agonist treatment. Instead, P2X_7_R mediated currents are incredibly increasing in amplitude upon repeated brief applications or sustained application of agonists [14, 15].

Although a growing number of studies show the involvement of P2X_7_R in the tumorigenesis, its role is still controversial. Previous studies have shown that activation of P2X_7_R can induce angiogenesis, increase vascular endothelial growth factor production, accelerate cell invasion and migration, and hence promote tumor growth [16, 17]. Recent evidences show a possible direct antiangiogenic role of P2X_7_R on tumor-derived endothelial cells [18]. However, other studies showed that inhibiting the activation of P2X_7_R induced tumor growth and accelerated tumor cell death [19, 20]. Thus, the role of P2X_7_R in glioma cell proliferation and migration remains unclear.

Herein, we examined the role of P2X_7_R on the migration, cell proliferation, and downstream signaling pathways using human glioma cell lines and human glioma tissues.

## 2. Materials and Methods

### 2.1. Human Glioma Samples

Twenty primary glioma and adjacent normal tissues were obtained from patients with glioma grades I to IV (5 samples per grade), who underwent surgical resection at the Affiliated Hospital of Nantong University. The study was approved by the Ethics Committee of the Affiliated People's Hospital of Jiangsu University and Affiliated Hospital of Nantong University. The clinical and pathological features of the patients were independently diagnosed by two independent pathologists. Every sample were collected and fixed in 10% formalin overnight and then dehydrated through gradient alcohol and xylenes. The samples were imbedded in paraffin and sectioned in 5 *μ*m thickness.

### 2.2. Culture of Human Glioma Cell Lines

The immortalized human malignant glioma cell lines U87 and U251 were purchased from Chinese Academy of Sciences Cell Bank (Shanghai, China) and were incubated in Dulbecco's modified Eagle's medium (Gibco, USA) supplemented with 10% fetal bovine serum (Gibco, USA) and antibiotics (100 U/ml penicillin and 100 *μ*g/ml streptomycin). The cell lines were maintained in a CO_2_ incubator containing 5% CO_2_ at 37°C and were used no more than F10.

### 2.3. Immunofluorescent Staining

Paraffin sections of human glioma tissue were deparaffinized, rehydrated, and blocked by 5% BSA in phosphate buffer saline (PBS) for 2 hours in room temperature. Sections were incubated with the primary antibody against P2X_7_R (1 : 400, Abcam, Cambridge, MA) at 4°C overnight. Then a second antibody (1 : 500, Jackson ImmunoResearch, West Grove, PA) and 4,6-diaminodiphenyl-2-phenylindole (DAPI, Sigma- Aldrich) were added in a dark room and incubated for 2-3 h. After washing, sections were mounted in 50% glycerol in PBS. The immunofluorescent signal of P2X_7_R was visualized under a fluorescent microscope (Leica, Germany).

U87 and U251 glioma cells were washed in PBS and then fixed in 4% paraformaldehyde solution for 20 min at room temperature. Cells were rinsed in PBS and incubated in PBS containing 0.1% Triton X-100 and 3% bovine serum albumin (BSA) for 1 hour to block the nonspecific binding sites. Then cells were incubated with antibody against P2X_7_R (1 : 400, Abcam, Cambridge, MA) at 4°C overnight. On the following morning, the appropriate secondary antibodies and DAPI were added in a dark room and incubated for 2-3 h. Each immunolabeling experiment was triplicates. After washing, cells were mounted, and the immunofluorescent signal was visualized under a fluorescent microscope (Leica, Germany).

### 2.4. 3-(4,5-Dimethylthiazol-2-yl)-2,5-diphenyltetrazolium Bromide (MTT) Assay

Proliferation of glioma cell lines was determined by MTT method. U87 and U251 glioma cells were seeded at a density of 1 × 10^5^ per well of 24-well plate and cultured overnight. The medium was replaced with fresh medium supplemented with different concentrations (5, 10, 50, 100, 500, and 1000 *μ*M) of BzATP. Before the end of treatment, 25 *μ*L of MTT solution (5 mg/ml) was added to cell cultures and incubated at 37°C for 3 hours. Then the medium was collected and formazan crystals were dissolved in 150 *μ*L dimethyl sulfoxide. Absorbance was measured at 570 nm with a Microplate Reader (Model 680, Bio-Rad, Hercules, CA). The value is shown as mean ± standard error (SE) from three independent experiments. Each experiment was performed in triplicate.

### 2.5. Western Blot Analysis

Western blot analysis was conducted as previously described [21]. Briefly, the lysates were boiled for 15 min followed by centrifugation at 12,000 rpm for 5 min, and the supernatant was collected. Protein concentrations were measured by Bio-Rad protein assay (Bio-Rad Laboratories, Segrate, Milan, Italy). Aliquots of lysates containing an equal amount of protein were resolved by 10% sodium dodecyl sulfate polyacrylamide gel electrophoresis (SDS-PAGE) and transferred to polyvinylidene difluoride (PVDF) membranes. The membranes were blocked with 5% skimmed milk at room temperature for 1 h followed by incubation with primary antibodies against P2X_7_R (1 : 1000, Abcam, Cambridge, MA), ERK (1 : 200, Cell Signaling Technology, Danvers, MA), p-ERK (1 : 200, Cell Signaling Technology, Danvers, MA), PCNA (1 : 200, Cell Signaling Technology, Danvers, MA), and glyceraldehyde 3-phosphate dehydrogenase (GAPDH, 1 : 1000, Sigma-Aldrich) at 4°C overnight. The PVDF membranes were then incubated with horseradish peroxidase- (HRP-) conjugated goat anti-rabbit or goat anti-mouse secondary antibody (1 : 2000, Thermo Scientific, Rockford, IL, USA) at room temperature for 2 hours. The membranes were incubated with chemifluorescent reagent ECL (Thermo Scientific, Rockford, IL, USA) and then exposed to X-ray film in the dark room. The protein bands were quantitatively analyzed with ImageJ software.

### 2.6. *In Vitro* Cell Migration Assay

U87 and U251 cell lines were cultured in 6-well plates till 70–80% confluency. The monolayer of cells was wounded with a sterile 200 *μ*l pipette tip in a straight line along the diameter of the well and then washed three times with PBS. The cells were cultured for further 24 h allowing cell migration into the open scratched area. Images of cells were captured at 0 and 24 hour after wounding, using a Leica microscope (Leica, Germany). The absolute value of distance migrated by cells was quantified as the change in the perpendicular distance between the edge of the gap after 24 hours. The value was then normalized to the 0 hour starting measurement, which represents “migration.”

### 2.7. siRNA Transfection

siRNA targeting specific sequences of P2X_7_R and a negative control (scrambled 1 siRNA) were synthesized by Gene Pharma Co. Ltd. (Shanghai, China). The siRNA sequences directed against P2X_7_R were sense: 5′-GGAUCCAGAGCAUGAAUUAUU-3′, antisense: 5′-UAAUUCAUGCUCUGGAUCCUU-3′. Transfections of control and P2X_7_R-siRNA were performed using Lipofectamine 2000 (Invitrogen) according to the manufacturer's instructions.

### 2.8. *In Situ* Terminal Deoxynucleotidyl Transferase dUTP Nick End Labeling (TUNEL) Staining

The malignant glioma cell lines U87 and U251 were seeded on cover glass which were placed in 24-well plates. TUNEL assay was performed at 24 hours after treatment with the BzATP using a fluorescein in situ cell death detection kit (Roche Applied Science, Germany) according to the manufacturer's instructions. Nuclei were stained with DAPI at room temperature for 15 min. The double-stained positive cells with DAPI and fluorescein were visualized under fluorescence microscope (Leica, Germany) and were quantified with Image J software.

### 2.9. Statistical Analysis

All experiments were independently repeated in triplicate. The value is presented as mean ± standard error. Statistical significance between groups was analyzed using *t*-test (two-sided, nonparametric) or one-way-ANOVA by GraphPad Prism. *P* value of less than 0.05 was considered statistically significant.

## 3. Results

### 3.1. P2X_7_R Expression in Human Glioma Cell

**Figure 1:**
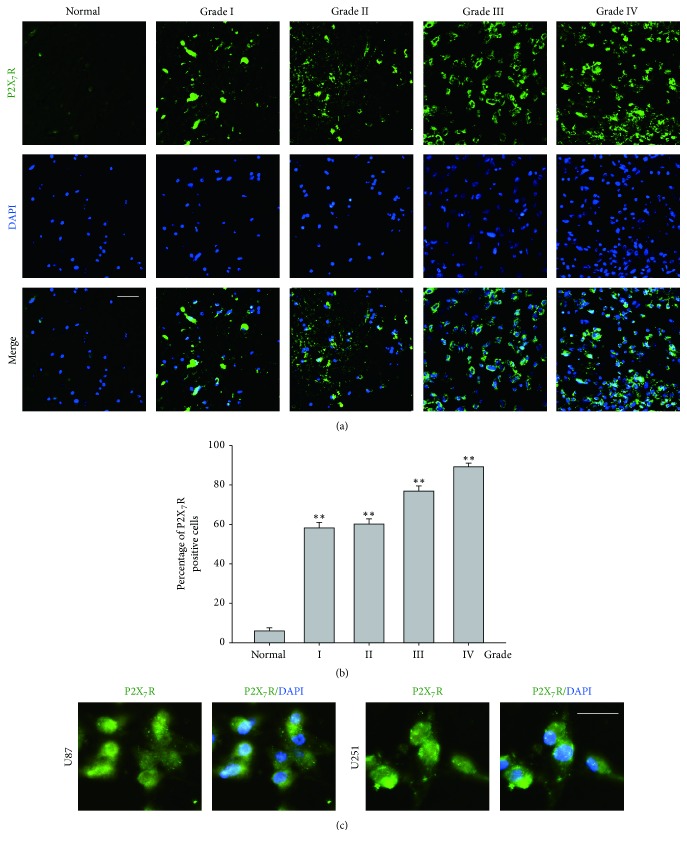
*P*2*X*_7_*R expression in different grades of human glioma and human glioma cells*. (a) Immunofluorescence labeling of P2X_7_R (green) in different grades of human glioma showed that P2X_7_R positive cells were rarely seen in normal brain tissue. As the glioma becomes more advanced, the percentage of P2X_7_ positive cells dramatically increased, especially in grade III and grade IV glioma. Scale bar = 50 *μ*m. (b) Bar charts showing the percentage of P2X_7_R positive cells in normal and different grades of glioma tissue (*n* = 5 for each group). ^*∗∗*^*P* < 0.01 versus Ctr. Data analyzed by ANOVA test. (c) Immunofluorescence labeling showing P2X_7_R protein expression in U87 cells and U251 cells. Scale bar = 20 *μ*m.

Paraffin sections of human glioma tissue with different stages of diagnosis or adjacent normal tissue were stained for P2X_7_R. We found that P2X_7_R positive glial cells were rarely seen in normal tissues. However, the P2X_7_R positive cell was detect to be increased in higher stage of glioma. The percentage of positive cell in normal tissue was 3.5 ± 0.6%, while the percentage of positive cell was 58.2 ± 2.1% in grade I (*P* < 0.01), 60.8 ± 1.9% in grade II (*P* < 0.01), 77.0 ± 1.9% in grade III (*P* < 0.01), and 89.3 ± 1.3% in grade IV (*P* < 0.01) (Figures 1(a) and 1(b)).

Other studies have also shown that most glioma cell lines expressed P2X_7_R [20, 22]. Here, we selected U87 and U251 glioma cell lines to check if these cell lines also express P2X_7_R. The immunofluorescence staining showed that P2X_7_R expressed in almost all cells in these two cell lines (Figure 1(c)).

### 3.2. BzATP Promotes the Proliferation and Migration of U87 and U251 Glioma Cells

**Figure 2:**
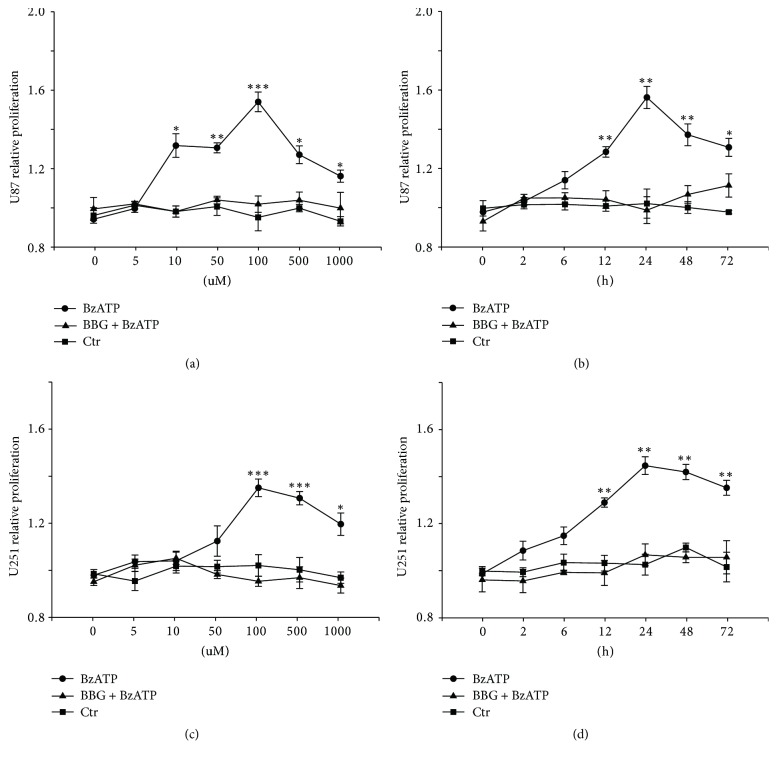
*BzATP promotes the proliferation of U87 and U251 glioma cells*. (a) The proliferation of U87 glioma cell with treatment of BzATP/BBG at different concentrations (5, 10, 50, 100, 500, and 1000 *μ*M). (b) The proliferation of U87 glioma cell with treatment of BzATP/BBG at different time points (2, 6, 12, 24, 48, and 72 h). (c) The proliferation of U251 glioma cell with treatment of BzATP/BBG at different concentrations (5, 10, 50, 100, 500, and 1000 *μ*M). (d) The proliferation of U251 glioma cell with treatment of BzATP/BBG at different time points (2, 6, 12, 24, 48, and 72 h). ^*∗*^*P* < 0.05, ^*∗∗*^*P* < 0.01, and ^*∗∗∗*^*P* < 0.001 compared to the control groups at the same time point.

MTT assay was performed to detect the effect of BzATP (5 to 1000 *μ*M) on glioma cell proliferation. We found that the proliferation of U87 and U251 glioma cell lines was significantly increased in the presence of 10–1000 uM and 100–1000 *μ*M BzATP, respectively. In addition, the peak of cell proliferation of both U87 and U251 cell lines was at 100 *μ*M BzATP (Figures 2(a) and 2(c)). To investigate the optimal incubation time of BzATP, both glioma cells lines were incubated with 100 *μ*M BzATP for 2 to 72 hours. We found that the optimal incubation time is 24 hours in both U87 and U251 cells lines (Figures 2(b) and 2(d)). The cell proliferation induced by BzATP could be blocked by brilliant blue G (BBG), the specific antagonist of P2X_7_R (Figures 2(a)–2(d)). These results suggest that BzATP leads to proliferation of glioma cell lines mediated by activation of P2X_7_R.

**Figure 3:**
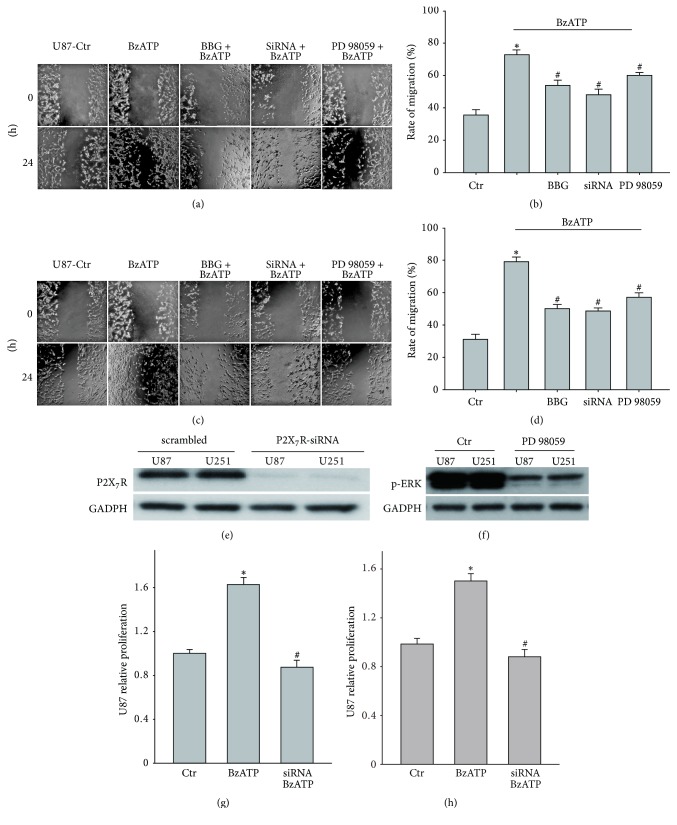
*BzATP enhances migration and proliferation of U87 and U251 glioma cells*. (a) Increased migration of the U87 cells with the treatment of BzATP and blocked by BBG (antagonist of P2X_7_R), P2X_7_R-siRNA, and PD98059 (ERK inhibitor) for 24 h. (b) Bar charts summarizing the migration of U87 cells under different conditions. (c) Increased migration of the U251 cells with the treatment of BzATP and blocked by BBG, P2X_7_R-siRNA and PD98059 for 24 h. (d) Bar charts summarizing the migration of U251 cells under different conditions. (e) A strong expression of P2X7R was detected in the scrambled siRNA treated groups and its expression was almost absent in the P2X_7_R-siRNA treated groups. (f) The expression of p-ERK was suppressed by PD98059. (g) The proliferation mediated by BzATP of U87 glioma cell was blocked by P2X_7_R-siRNA. (h) The proliferation mediated by BzATP of U251 glioma cell was blocked by P2X_7_R-siRNA. ^*∗*^*P* < 0.05 compared with the control; ^#^*P* < 0.05 compared with the BzATP group.

Next, we examined the effect of BzATP on the migration of glioma cells scratch injury. The migration rate of U87 cells in the untreated group was 39.7 ± 2.3% while BzATP (100 *μ*M, 24 h) treated group was 73.0 ± 2.1% (*P* < 0.05, *t*-test). The effect of BzATP was abolished by P2X_7_ antagonist BBG and P2X_7_R -siRNA with the migration rate 53.0 ± 6.6% (compare to BzATP group, *P* < 0.05, *t*-test) and 43 ± 5.7% (compare to BzATP group, *P* < 0.05, *t*-test). In addition, our results also showed that PD98059, the blocker of MEK/ERK pathway, partially blocked the BzATP-induced migration of U87 glioma cell lines (Figures 3(a) and 3(b)). Similar results were observed in the U251 glioma cell line (Figures 3(c) and 3(d)). P2X_7_R-siRNA almost completely blocked the expression of P2X_7_R in Western blot (Figure 3(e)). PD98059 had a high efficiency in suppressing the expression of p-ERK in Western blot (Figure 3(f)). We also checked the effect of P2X_7_R-siRNA on BzATP mediated cell proliferation. MTT data showed that the 24 hours incubation of 100 *μ*M BzATP increased the U87 and U251 cell proliferation to 1.6-fold compared to control group, while P2X_7_R-siRNA totally blocked the effect (Figures 3(g) and 3(h)). It suggests that activation of P2X_7_R enhances the migration and proliferation of human glioma cell lines.

**Figure 4:**
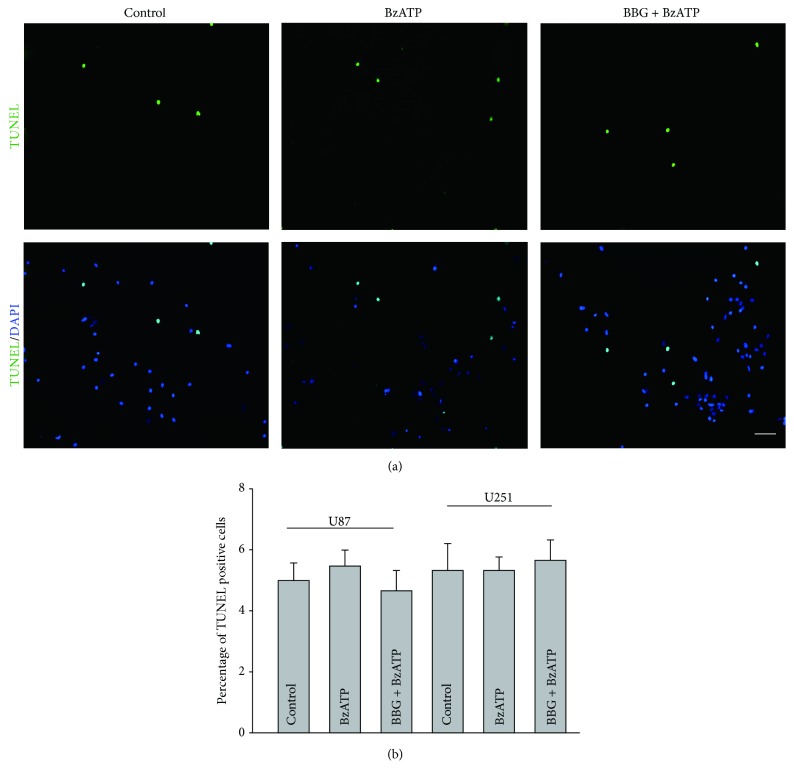
*BzATP does not enhance apoptosis in U87 and U251 glioma cells*. (a) Microphotographs show representative confocal images of TUNEL signals (green) and DAPI (blue) in U87 glioma cells treated by BzATP and blocked by BBG. (b) Bar charts summarizing the percentage of TUNEL positive cell in U87 and U251 cells treated by BzATP and blocked by BBG.

To investigate if BzATP affect the cell survival of glioma cell lines, we determine the number of apoptotic cells of U87 and U251 cell lines following 24-hour incubation of 100 *μ*M BzATP. TUNEL assay was used to detect the apoptosis of glioma cell lines. Compared to the control/untreated group, the number of TUNEL positive cells in the BzATP-treated group had no significant change (Figure 4).

**Figure 5:**
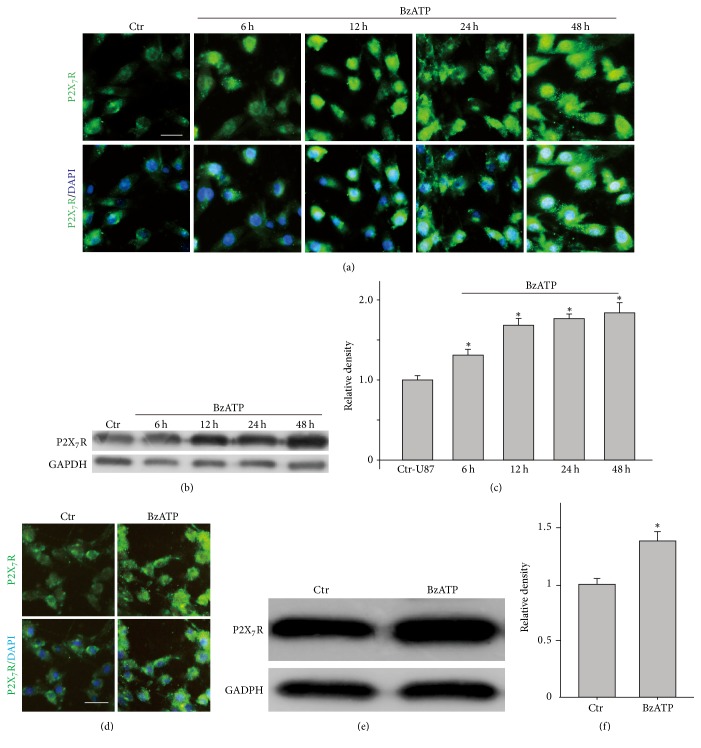
*P*2*X*_7_*R protein expression was induced by BzATP in human glioma cells*. (a) Immunofluorescence labeling showing P2X_7_R protein expression in control (Ctr) and BzATP (100 *μ*M)-treated U87 cells for different time (6–48 h). Scale bar = 20 *μ*m. (b) Representative immunoblots showing P2X_7_R protein expression in Ctr and BzATP (100 *μ*M)-treated U87 cells for different time (6–48 h). (c) Bar charts summarizing the average densitometric quantification of immunoreactive bands of P2X_7_R proteins of U87 cells under different conditions, *n* = 6 for each group. ^*∗*^*P* < 0.05 versus Ctr. (d) Immunofluorescence labeling showing the changes in P2X_7_R protein expression in Ctr and BzATP (100 *μ*M)-treated U251 cells for 24 h. Scale bar, 20 *μ*m for all images. (e) Representative immunoblots showing the changes in P2X_7_R protein expression in Ctr and BzATP (100 *μ*M)-treated U251 cells for 24 h. (f) Bar charts summarizing the average densitometric quantification of immunoreactive bands of P2X_7_R proteins of U 251 cells in Ctr and BzATP (100 *μ*M)-treated groups, *n* = 6 for each group. ^*∗*^*P* < 0.05 versus Ctr.

The cytotoxicity and necrosis of malignant glioma cells can increase the release of ATP and also its accumulation in the microenvironment of glioma tissue, while the capability of ATP degradation during the pathological condition was decreased [23]. Excessive extracellular ATP might lead to an activation and expression of P2X_7_R in human and rat glioma cells [22]. To investigate if P2X_7_ agonist BzATP could increase the expression of P2X_7_R in U87 and U251 glioma cell lines, immunocytochemical staining and Western blot were used to determine the changes of P2X_7_R expression. We found that BzATP induced the upregulation of P2X_7_R in U87 and U251 glioma cells (Figure 5).

### 3.3. Involvement of MEK/ERK Pathway in BzATP Mediated Proliferation of U87 and U251 Glioma Cells

**Figure 6:**
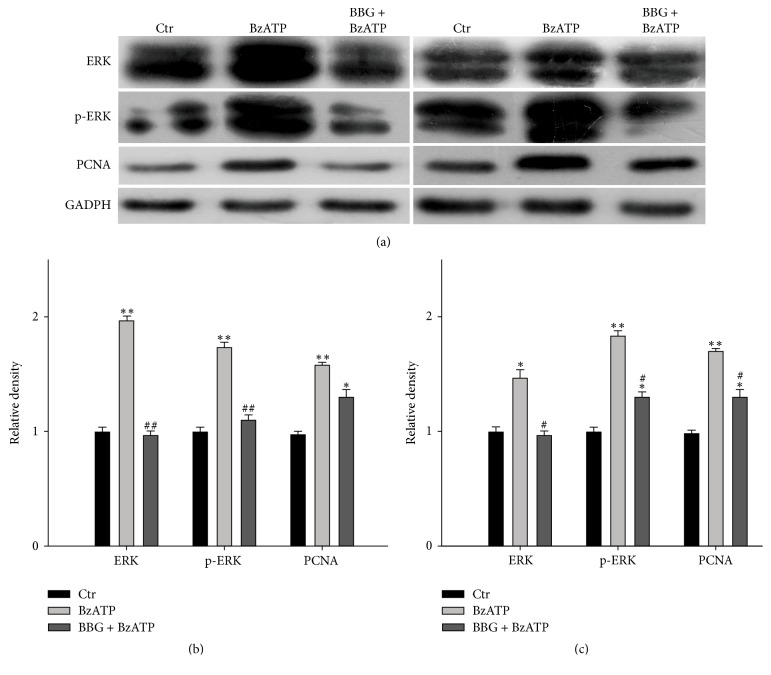
*BzATP-induced activation of ERK pathway in U87 and U251 glioma cells*. (a) Representative immunoblots showing the changes in ERK, p- ERK, and PCNA protein expression in control (Ctr), BzATP (100 *μ*M), and BzATP + BBG-treated U87 (left) and U251 (right) cells. (b, c) Bar charts summarizing the average densitometric quantification of immunoreactive bands of ERK, p-ERK, and PCNA proteins of U87 (b) and U251. (c) Cells under different conditions, *n* = 6 for each group. ^*∗*^*P* < 0.05 and ^*∗∗*^*P* < 0.01 versus Ctr; ^#^*P* < 0.05 and ^##^*P* < 0.01 versus BzATP group.

MEK/ERK pathway is a common intracellular signaling pathway related to glioma cell proliferation [24]. Our study also demonstrated the role of MEK/ERK pathway in the proliferation and migration of glioma cells induced by P2X_7_R activation. Proliferating cell nuclear antigen (PCNA) is only found in normal proliferating cells and tumor cells. In general, the expression level of PCNA in tumor is correlated with the degree of malignancy. Here, we first detected the expression of ERK/p-ERK protein with activation of P2X_7_R. The results showed that BzATP significantly increased of ERK, p-ERK, and PCNA protein expression in both U87 and U251 cell lines. This effect was completely abolished in the presence of BBG (Figure 6).

**Figure 7:**
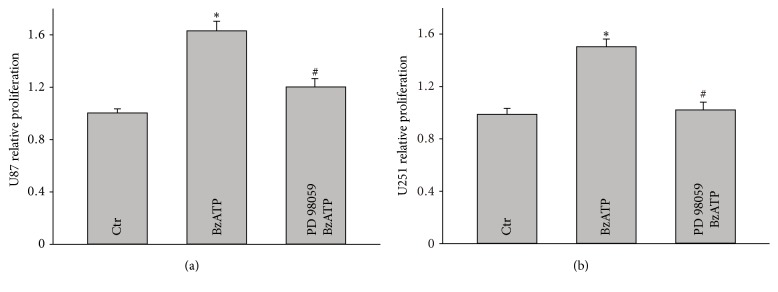
*Involvement of ERK pathway in the BzATP mediated proliferation of U87 and U251 glioma cells*. (a) The proliferation of U87 glioma cell upon treatment of BzATP (100 *μ*M) alone or together with PD98059 for 24 h. (b) The proliferation of U251 glioma cell upon treatment of BzATP (100 *μ*M) alone or together with PD98059 for 24 h; ^*∗*^*P* < 0.05 compared to the control groups; ^#^*P* < 0.05 compared to the BzATP groups.

We further investigated if BzATP induced glioma cell proliferation and migration are mediated by ERK pathway. Results showed that PD98059, the specific inhibitor of MEK/ERK pathway, completely inhibited the BzATP-induced proliferation of glioma cells in U87 and U251 cell lines (Figures 7(a) and 7(b)). Overall, these results suggest that the MEK/ERK pathway plays an important role in glioma cell proliferation and migration mediated by the activation of P2X_7_R.

## 4. Discussion

### 4.1. Activation of P2X_7_R Induces Proliferation and Migration of Glioma Cells

Microenvironment of tumors including glioma is characterized by a strikingly high concentration of adenosine and ATP [6]. P2X_7_R is an ATP-gated cation channel that regulates cell proliferation and apoptosis [25–28] and it is widely expressed in the immune system and nervous system [28, 29]. P2X_7_R expression would increase in various inflammatory diseases, neurodegenerative diseases, neuropathic pain, and trauma [29–31]. In addition, it is also expressed in different types of tumors such as leukemia, prostate cancer, breast cancer, neuroblastoma, and thyroid papillary carcinoma [32–34]. Some studies have reported that P2X_7_R activation correlated with tumor severity, prognosis, and survival. For example, in breast tumor, P2X_7_R activation promoted tumor cell proliferation, while KN62, the P2X_7_R antagonist, or shRNA of P2X_7_R inhibited the proliferation and even promoted the apoptosis of tumor cells [34]. The metastatic ability of lymphoma cells decreased significantly while P2X_7_R was silenced by shRNA [34]. Giannuzzo et al. found that P2X_7_R were expressed in human pancreatic cancer cells, and AZ10606120, a specific inhibitor of P2X_7_R, inhibited the metastasis and invasion of pancreatic tumor cells [35]. In neuroblastoma, increased expression and activation of P2X_7_R accelerate the proliferation and metastasis of tumor cells. Higher percentage of P2X_7_R positive tumor cells makes poor prognosis of neuroblastoma [36]. In present study, P2X_7_R expression increased with a higher grade of gliomas, suggesting that P2X_7_R expression was associated with the tumor prognosis. The increasing expression of P2X_7_R in highest grades of glioma tissues could be a secondary effect of the tumor progression and not necessarily a causal factor. Our in vitro study showed that activation of P2X_7_R promoted proliferation and migration of human glioma cells, and the effects were blocked by an antagonist of the receptor. Targeting P2X_7_R seems to have a suppression effect on glioma progression.

### 4.2. P2X_7_R Activation Does Not Induce Apoptosis of Glioma Cells

Brief stimulation of P2X_7_R leads to increase of intracellular calcium influx whereas repeated or prolonged stimulation of P2X_7_R induces the formation of a nonselective pore allowing the entry of solutes up to 900 Da in size, which leads to membrane blebbing, release of cytokines, and eventually cell death [14, 15]. Thus, overactivation of P2X_7_R can lead to different kinds of cell death [9]. In some neurons, activation of P2X_7_R leading to cell death is one of the key mechanisms causing neurodegeneration in Alzheimer's disease and multiple sclerosis [37, 38]. Our previous data also showed activation of P2X_7_R increased the death of retinal ganglion cell in glaucoma [21, 39]. However, in tumor tissues, P2X_7_R activation inhibited the apoptosis of tumor cells and promoted cell proliferation [40]. A number of studies have reported that elevated levels of extracellular ATP contribute to the progression of brain tumor growth that may be correlated with the activation of P2X_7_R [41–43]. Despite considerable technical difficulties of measuring extracellular ATP concentration, it is estimated that extracellular ATP in millimolar level could be present in pathological condition of inflammation and tumor [44]. In human glioma cell lines U87 and U251, it was reported that ATP in millimolar concentration promoted the release of cytokines such as IL-8 without cells death [22]. In our present study, BzATP which has a higher affinity than ATP to P2X_7_R was used to activate P2X_7_R. Although other studies showed BzATP could cause the formation of large pore size and cell death in human and rat central nervous system [9], the present study showed no significant increase in apoptosis of BzATP-treated human glioma cells lines.

### 4.3. The MEK/ERK Pathway Was Involved in P2X_7_R Mediated Proliferation and Migration of Glioma Cells

The highly conserved RAS-mitogen activated protein kinase (MAPK) signaling pathway is involved in a wide range of cellular processes, including cell survival, differentiation, and proliferation [45]. MEK/ERK pathway is one of the major pathways in MAPK pathway. In tumor cells, members of the MEK/ERK pathway encoding genes are often mutated and become overactivated, which causes this pathway to be important for the development of many human tumors such as breast cancer, thyroid carcinoma, and squamous cell carcinoma [46, 47]. The MEK/ERK pathway also plays a critical role in the development of gliomas [24, 48, 49]. The MEK/ERK pathway is involved in purinergic receptor, such as P2X_7_R, mediated excitotoxic neuronal injury, and neuroprotection [50]. Our results showed that the ERK/p-ERK and PCNA protein expressions were increased in BzATP-treated glioma cell lines. As P2X_7_R is a nonspecific cation channel receptor, its activation will increase the intracellular calcium concentration and finally cause the expression of ERK and its phosphorylation. We further found that P2X_7_R mediated glioma cell proliferation and migration dramatically decreased by blocking the MEK/ERK pathway. Therefore, our results suggest that MEK/ERK pathway is involved in P2X_7_R mediated proliferation and migration of human glioma cells.

In conclusion, we found that the number of P2X_7_R positive glioma cells increased with the grade of human tumor. Activation of P2X_7_R in human glioma cells in vitro promoted the proliferation and migration of glioma cells but has no significant effect on apoptosis. Using a specific MEK blocker PD98059, we revealed that MEK/ERK pathway was involved in the P2X_7_R mediated the proliferation and migration of glioma cells. As P2X_7_R inhibitors have been tested in clinical trials, our results supports the idea that blocking the P2X_7_R activation may be a feasible approach to prevent the progression of glioma.
